# Morphometric similarity differences in drug‐naive Parkinson's disease correlate with transcriptomic signatures

**DOI:** 10.1111/cns.14680

**Published:** 2024-03-26

**Authors:** Yajie Wang, Yiwen Xiao, Yi Xing, Miao Yu, Xiao Wang, Jingru Ren, Weiguo Liu, Yuan Zhong

**Affiliations:** ^1^ Department of Neurology The Affiliated Brain Hospital of Nanjing Medical University Nanjing China; ^2^ Department of Neurology The First People's Hospital of Yancheng Yancheng China; ^3^ School of Psychology Nanjing Normal University Nanjing China; ^4^ Department of Radiology The Affiliated Brain Hospital of Nanjing Medical University Nanjing China

**Keywords:** Allen human brain atlas, morphometric similarity, Parkinson's disease, transcriptomic

## Abstract

**Background:**

Differences in cortical morphology have been reported in individuals with Parkinson's disease (PD). However, the pathophysiological mechanism of transcriptomic vulnerability in local brain regions remains unclear.

**Objective:**

This study aimed to characterize the morphometric changes of brain regions in early drug‐naive PD patients and uncover the brain‐wide gene expression correlates.

**Methods:**

The morphometric similarity (MS) network analysis was used to quantify the interregional structural similarity from multiple magnetic resonance imaging anatomical indices measured in each brain region of 170 early drug‐naive PD patients and 123 controls. Then, we applied partial least squares regression to determine the relationship between regional changes in MS and spatial transcriptional signatures from the Allen Human Brain Atlas dataset, and identified the specific genes related to MS differences in PD. We further investigated the biological processes by which the PD‐related genes were enriched and the cellular characterization of these genes.

**Results:**

Our results showed that MS was mainly decreased in cingulate, frontal, and temporal cortical areas and increased in parietal and occipital cortical areas in early drug‐naive PD patients. In addition, genes whose expression patterns were associated with regional MS changes in PD were involved in astrocytes, excitatory, and inhibitory neurons and were functionally enriched in neuron‐specific biological processes related to trans‐synaptic signaling and nervous system development.

**Conclusions:**

These findings advance our understanding of the microscale genetic and cellular mechanisms driving macroscale morphological abnormalities in early drug‐naive PD patients and provide potential targets for future therapeutic trials.

## Graphical abstract

Overview of the study methodology. Morphometric similarity (MS) network analysis was used to quantify the interregional structural similarity from multiple MRI anatomical indices measured at each brain region of early drug‐naive PD patients.

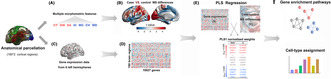

## 1 INTRODUCTION

Parkinson's disease (PD) is a common neurodegenerative disease characterized by the intracellular aggregation of alpha‐synuclein, which disrupts the normal neural circuit throughout the brain and produces heterogeneous motor and non‐motor symptoms.^1^, ^2^ Although previous studies have demonstrated that the spread of pathologically toxic proteins is related to genetic factors and anatomical connectivity,^3^, ^4^, ^5^ the pathophysiological mechanism of local brain region transcriptomic vulnerability remains unclear. In addition, pathological transmission and microstructural damage driven by genetic factors occur years to decades before clinical symptom onset, and further deterioration occurs due to the combined effects of the disease's course, environment, and drug treatment.^1^, ^6^, ^7^ Therefore, it is crucial to understand how differences in regional gene expression drive selective vulnerability for structural changes in early drug‐naive PD patients.

Previous magnetic resonance imaging (MRI) studies of PD have indicated progressive cortical atrophy accumulation in the frontal, parietal, temporal, and hippocampal areas and in the basal ganglia.^8^, ^9^ However, the atrophy of the cortex and subcortical nuclei in PD patients is subtle and regionally nonspecific, especially during relatively early‐stage PD.^10^, ^11^ Rather than simply describing this as heterogeneous, distributed cortical atrophy, accumulating evidence from neuropathological and neuroimaging studies indicates that PD is considered a structural and functional “disconnection” syndrome.^5^, ^7^, ^12^ A longitudinal study showed that decreased connectivity between regions was initially found in parahippocampal and temporal areas in early drug‐naive PD patients; with disease progression, this effect evolved into a more widespread cortical pattern that was correlated with clinical motor and cognitive abnormalities.^13^ Another neuroimaging study revealed that functional connectivity was commensurately increased in PD patients taking dopaminergic medication compared to drug‐naive patients.^14^ In addition to dopaminergic drugs, other therapeutic agents have been reported to induce significant changes in intrinsic connectivity between brain areas.^15^ Therefore, exploring the brain connectome of early drug‐naive PD patients more could directly reflect the disease‐related brain structure changes and rule out potentially confounding effects of chronic disease duration and medication. To investigate the brain connectome dysfunction in PD, previous studies have mainly used tractographic analysis of diffusion weighted imaging (DWI) and structural covariance analysis of morphological measures.^5^, ^7^ However, the application of DWI‐based tractography remains limited in estimating the long‐distance anatomical connections, for example, between bilateral homologous areas of the cortex. Structural covariance analysis is used to construct intersubject covariance networks based on a certain morphological indicator of a group of subjects. This conventional morphological network is not applicable to individual analysis, and its biological interpretation is controversial.^16^, ^17^, ^18^ Recently, morphometric similarity (MS) network analysis has been used to quantify the interregional structural similarity from multiple MRI anatomical indices measured at each brain region in individuals.^19^ Based on the histological theory that cortex regions with similar cytoarchitectonic classes are more likely to be connected anatomically and have high levels of co‐expressed genes enriched for neuronal terms,^20^, ^21^ MS network analysis could be used to build a more closely connected whole‐brain anatomical network at the microstructural level than DWI‐based tractography and structural covariance analysis. MS network analysis has been used to explore the correlation between brain structural changes and underlying transcriptomic signatures for various mental and neurological diseases.^22^, ^23^, ^24^, ^25^ However, this novel approach has not been applied to PD patients, particularly early drug‐naive PD patients, which could avoid the potential confounding influence of medication and disease duration.

Current studies have combined neuroimaging data with gene expression data from available human post‐mortem brains to investigate regional differences in disease‐related gene expression and gain further insight into the underlying molecular mechanisms of brain morphological changes in various neuropsychiatric disorders.^3^, ^24^, ^26^, ^27^, ^28^ The Allen Human Brain Atlas (AHBA) consists of microarray data measured from 3702 spatially distinct samples taken from six adult brains without any history of neurological disorders, thus providing a high‐resolution gene expression map of the whole brain.^29^ In the past few years, morphological studies utilizing AHBA data have demonstrated that regional brain atrophy and white matter connectivity loss in PD are associated with spatial gene expression.^4^, ^30^ Using a similar approach, a recent study identified that spatial brain iron accumulation measured by quantitative susceptibility mapping (QSM) is related to regional transcriptome patterns, driving regional selective vulnerability to neurodegeneration.^31^ Moreover, studies have explored the biological and cellular processes, indicating the significant enrichment of glutamatergic and GABAergic neurons in PD.^5^, ^31^ Thus, transcription‐neuroimaging association analyses can provide an unprecedented chance to further uncover the molecular mechanism of macroscale brain abnormalities in PD.

Here, we combined the spatial morphometric information measured using multiple structural MRI with regional transcriptional signatures from the AHBA dataset to shed light on the underlying mechanisms of regional selective vulnerability in early‐stage PD. We first used partial least squares (PLS) regression to identify specific gene expression patterns spatially related to regional MS changes in PD. Then, we obtained the PD‐related genes and correlated them with differentially expressed genes in post‐mortem brain tissue from patients with PD and other degenerative diseases. Moreover, we also focused on the biological processes in which the PD‐related genes were enriched and the cellular characterization of these genes to understand how disease‐related genetics drive the morphological phenotype in PD.

## 2 MATERIALS AND METHODS

### 2.1 Subjects

One hundred and eighty drug‐naive PD patients and sex‐ and age‐matched 132 healthy controls (HCs) were recruited from the Affiliated Brain Hospital of Nanjing Medical University. Ten PD patients and nine HCs were excluded due to poor imaging quality. The data of 170 PD patients and 123 HCs were finally analyzed. The inclusion criteria for the PD patients were as follows: (1) 40–80 years old; (2) PD diagnosis as per the United Kingdom PD Society Brain Bank Criteria; (3) no history of taking anti‐PD drugs; and (4) clinical follow‐up continued for at least 1 year. The HCs were neurologically normal. The exclusion criteria for all participants were as follows: (1) history of serious diseases that could influence cerebral function, such as brain damage, cerebrovascular disease, white matter disease, and brain operation; (2) history of serious diseases, such as cancer, diabetes, hyperthyroidism, severe psychiatric, and systemic illnesses; (3) dementia or inability to complete neuropsychological tests or an MRI scan; and (4) medication, such as anticholinergic drugs or antidepressants. All participants underwent clinical and MRI assessments.

The motor symptoms of PD patients were assessed using the motor subscale of the Unified Parkinson's Disease Rating Scale (UPDRS part III) and Hoehn and Yahr (H–Y) stages. The Hamilton Depression Rating Scale (HAMD) and the Hamilton Anxiety Scale (HAMA) were used to quantify depression and anxiety, respectively. The Mini Mental State Examination (MMSE) and the Montreal Cognitive Assessment (MoCA) were used to assess global cognitive function. The Medical Ethics Committee of the Affiliated Brain Hospital of Nanjing Medical University approved the study, and informed consent was obtained from all subjects.

### 2.2 Statistical analysis

The Kolmogorov–Smirnov (*n* > 50) test was employed to assess the normal distribution of the data. Continuous variables were expressed as means ± standard deviations (SDs) and categorical variables as numbers (%). Demographic and clinical variables were compared between the HC and PD groups. The two‐sample *t*‐test helped compare the quantitative variables that obey the normal distribution. Moreover, not normally distributed quantitative variables were compared with the Mann–Whitney *U*‐test. The chi‐square test helped analyze the qualitative variables. Pearson's correlation analysis helped analyze the correlation between MS values of significant abnormal regions and clinical features in PD patients. FDR correction was used to assess multiple comparisons. Statistical analyses were performed using SPSS version 26.0 (SPSS, Inc.), and figures were created in GraphPad Prism version 8.3.0 (GraphPad Software) and OriginPro 2021 version 9.8.0.200 (OriginLab). The brain maps were presented using Surf Ice (v1.0.20190902, https://www.nitrc.org/projects/surfice/).

### 2.3 MRI data acquisition and preprocessing

All participants underwent a brain structural T1 image with a 3T Verio Siemens scanner. T1‐weighted images for image registration and functional localization were acquired by 3D spoiled gradient echo (SPGR), and the parameters were the following: repetition time (TR) = 2530 ms; echo time (TE) = 3.34 ms; flip angle (FA) = 7 degrees; number of slices = 128; slice thickness = 1.33 mm; matrix = 256 × 192; field of view FOV = 256 × 256 mm.

The structural T1 images were analyzed using FreeSurfer (version 6.0.0, http://surfer.nmr.mgh.harvard.edu) to perform cortical modeling and volumetric segmentation and to measure multiple structural indices. The process included (1) correction of signal intensity nonuniformities; (2) removal of the skull; (3) affine registration to the Talairach atlas and segmentation of gray/white matter; (4) tessellation of the gray‐to‐white and gray‐to‐CSF borders; (5) automatic correction of topology defects; (6) surface reconstruction and smoothing; (7) surface inflation and registration to a spherical atlas for intersubject matching of cortical folding patterns; and (8) parcellation of the cortical mantle into the 360 brain regions of the Human Connectome Project (HCP) atlas. All images were carefully checked for inaccuracy and manually corrected as needed.

### 2.4 Structural network construction

**FIGURE 1:**
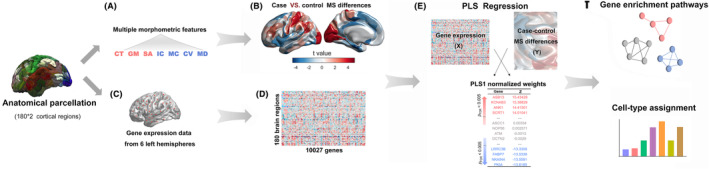
Overview of the study methodology. (A) The cortex was divided into 360 regions by the Human Connectome Project (HCP) atlas, and then seven morphometric features (e.g., cortical thickness, gray matter volume, surface area, Gaussian curvature, mean curvature, curved index, and folding index) were extracted from each of the T1W images to construct the morphometric similarity network (MSN). (B) The *t* statistics were mapped for the case‐control differences in regional MS. (C and D) Gene expression data from six left hemispheres from the Allen human brain atlas (AHBA) were mapped to 180 regions and were used to create a matrix containing 10,027 gene expression levels in those regions. (E) Partial least squares (PLS) regression was used to determine the correlation between regional MS changes and 10,027 gene transcripts in the left hemisphere, and then we obtained the first component of the PLS (PLS1). (F) Analyses of enrichment pathways and cell‐type‐specificity were performed on the PLS1 gene list.

The HCP divided the cortex into 360 regions through a sharp change in structure and connection, 180 in each hemisphere.^32^ The HCP atlas was assigned to the cortical surface of each participant, and subsequently, seven feature values were extracted from each of the T1W images. The values included cortical thickness (CT), gray matter volume (GM), surface area (SA), Gaussian curvature (IC), mean curvature (MC), curved index (CV), and folding index (FD). After Z‐normalization,^19^ the morphometric feature vector for each participant was used to form the MS network construction (no thresholding) by performing Pearson's correlation analysis (see Figure 1 for an overview of the MS network and gene expression processing).

### 2.5 Case‐control morphometric similarity analysis

Regional MS values were obtained by weighting the values of each region in a 360 × 360 MS network and adding them to the relevant values of other regions. Using the regional MS values as the dependent variable, the linear regression model (LRM) was employed to determine case‐control differences. Gender and age were set as covariates. The model was applied to all regions, and the two‐sided *t*‐values (contrast = PD − HC) were obtained. A model was developed to compare case‐control MS values by region (MS_*i*_): MS_*i*_ = intercept + *β*_1_ × (gender) + *β*_2_ × (age) + *β*_3_ × (group). Pearson's correlation analysis was used to analyze the correlation between the mean control regional MS and the *t* statistics for the case‐control differences in regional MS.

### 2.6 Spatial correlation between MS and gene expression

The Allen human brain atlas (AHBA) provided normalized microarray gene expression data based on the HCP atlas. However, there are two sets of gene data for the right hemisphere provided by AHBA (https://human.brain‐map.org), only the data for the left hemisphere were considered in the subsequent analysis. The AHBA consists of expression measurements for more than 20,000 genes measured from 3702 spatially distinct tissue samples taken from six adult brains. The AHBA dataset was processed using the method proposed by Arnatkeviciute et al.^29^ The process included: (1) using the re‐annotator toolkit to verify the annotations of probe‐to‐gene; (2) filtering the probe that does not exceed background noise; (3) selecting the representative probes to index expression for a gene; (4) mapping the tissue samples from the AHBA to the HCP 360 atlas; (5) using a scaled robust sigmoid of participants to normalize the expression measures; (6) filtering the inconsistently expressed. Finally, we obtained a total of 10,027 background genes (after intensity‐based filtering and probe selection based on correlation to RNA‐seq data). The transcript‐level matrix derived from the analysis included 10,027 gene expression levels in 180 regions. The correlation between regional variation in MS and 10,027 gene transcripts was determined by partial least squares regression (PLS), with gene expression results serving as predictor variables. The first component of the PLS (PLS1) was a linear combination of the gene expression values most associated with regional variation in MS. A permutation test based on spherical rotation (5000 rotations) was used to test the validity of PLS1.^33^ Bootstrapping estimated PLS1 variability for each gene, and the Z‐score was calculated by normalizing the weight of each gene with the standard error of Bootstrap.^22^ Genes associated with regional variation were selected by Z‐score (PLS1+, Z > 5; PLS1−, Z < −5. *p*_FDR_ < 0.005).

### 2.7 PD‐related genes from AHBA dataset analysis

In total, 107 genes were identified by searching “Parkinson disease” under “Gene Classifications” in the AHBA dataset (https://human.brain‐map.org/microarray/search). Then, we defined 64 PD‐related genes after filtering based on differential stability by overlapping with 10,027 background genes. These genes are known to be associated with the physiological processes of PD. To explore the contribution of PD‐related genes in the PLS analysis, overlapping genes were first obtained from the list of 64 PD‐related genes and 2022 PLS1 genes. Pearson's correlation analysis was then used to assess the relationship between overlapping gene expression and case‐control MS changes in the left hemisphere. Significant was set at *p* < 0.05, with FDR correction for multiple comparisons.

### 2.8 Correlation between PLS1 genes and differentially expressed genes in post‐mortem

To examine whether the PD‐related differentially expressed genes (DEGs) in post‐mortem brains are dysregulated in cortical regions with MS changes related to PD. We further compared the PLS1− genes related to the MS changes in PD with the DEGs from post‐mortem brains with synucleinopathies including PD (samples from cortical and subcortical brain regions), dementia with Lewy bodies (DLB), and multiple system atrophy (MSA). The PD‐related DEG list included 536 upregulated and 849 downregulated (*p* < 0.001) genes in the brain cortex of PD patients, as reported by Riley et al.^34^ Additionally, the gene list included 5314 upregulated and 455 downregulated genes in the striatum, as well as 539 upregulated and 2422 downregulated genes in the substantia nigra of PD patients. Pearson's correlation analysis was performed to assess the relationship between PLS1‐gene weights and upregulated or downregulated DEGs values (*p* < 0.05, FDR‐corrected). The above‐mentioned correlation analysis was also applied to the other synucleinopathies, including DLB and MSA^35^ (*p* < 0.05, FDR‐corrected).

### 2.9 Enrichment analysis

The automated meta‐analysis tool Metascape (https://metascape.org/) was used to elucidate the pathways of gene involvement. The identification of consistent potential pathways or networks among multiple gene lists is critical.^36^ Therefore, a list of PLS1− genes from PD patients was submitted to the Metascape website to compare the genetic identity and ontology of PLS1− with GWAS genes. Selected databases included the Gene ontology (GO) for biological processes and the Kyoto Encyclopedia of Genes and Genomes (KEGG). The significance threshold of the obtained enrichment pathways was 5%, corrected by FDR.

### 2.10 Cell type assignment to PLS1 genes

Data from five different single‐cell studies were compiled using postmortem cortical samples.^23^ The list of cell‐specific gene sets compiled from all available large‐scale single‐cell studies of adult human cortex was obtained from Seidlitz et al.^37^ (https://staticcontent.springer.com/esm/art%3A10.1038%2Fs41467‐020‐17051‐5/MediaObjects/41467_2020_17051_MOESM8_ESM.xlsx). Cells were classified into seven types, namely astrocytes, endothelial cells, microglia, excitatory neurons, inhibitory neurons, oligodendrocytes, and oligodendrocyte precursors (OPCs). The *p*‐values for the number of overlapping genes in each cell type were obtained by the permutation test (*p* < 0.05, FDR‐corrected). The aim was to identify the cell types to which these genes could be attributed.

## 3 RESULTS

### 3.1 Samples

The data of 170 PD patients and 123 HCs were finally analyzed after excluding 10 patients and 9 HCs due to excessive head motion. The Euler number, which quantifies image quality,^38^ was not significantly different between the two groups in our study (*t* = 0.10, *p* = 0.96; Supplemental Result S1 and Figure S1 in Data S1). The demographic and clinical characteristics of all subjects were summarized in Table S1. There were no significant differences between the two groups regarding age or sex. Compared to the controls, the PD patients had more years of education and higher MMSE and HAMD scores (all *p* < 0.05).

### 3.2 Case‐control differences in morphometric similarity

**FIGURE 2:**
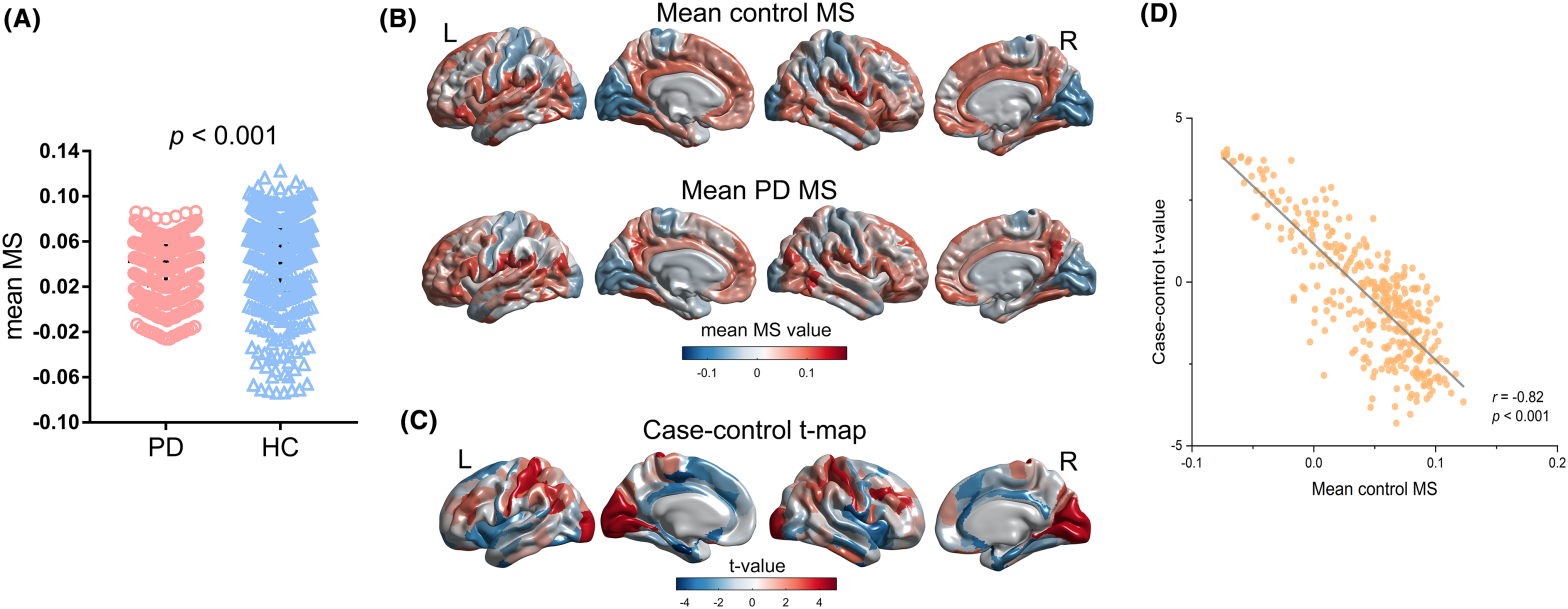
Case‐control differences in regional morphometric similarities. (A) The distributions of mean regional MS values in controls and PD patients. (B) The cortical map of the mean MS values in controls and PD patients. (C) The *t* statistics (*t*‐map) of the case‐control MS differences in regions. (D) The mean control regional MS was negatively correlated with the *t* statistics for the case‐control differences in regional MS (Pearson's *r* = −0.82, *p* < 0.001).

Globally, morphometric similarity was reduced in PD patients compared to controls (Figure 2A). The cortical maps of regional MS showed the anatomical distribution of positive and negative similar regions in the PD and HC groups (Figure 2B). The anatomical distribution of positive and negative MS strength for brain regions in the controls was consistent with that of prior reports.^19^, ^22^ This replicable pattern of regional MS in healthy individuals further confirmed the prior knowledge that the primary cortex is more histologically differentiated than the associated cortex. In addition, we mapped the *t* statistics for the case‐control differences in regional MS at each cortical region (Figure 2C). The positive or negative t statistic signified increased or decreased MS, respectively, in PD patients compared to controls. Compared to the HC group, the PD group showed significantly decreased regional MS weights in the primary, early visual, somatosensory, superior parietal, inferior parietal, and dorsolateral prefrontal cortices and increased MS weights in the posterior, insular and frontal opercular, mid cingulated, medial and lateral temporal, anterior cingulate, and medial prefrontal cortices (all *p*_FDR_ < 0.05; Supplemental Result S2 and Table S2 in Data S1). Decreased regional MS in PD patients means that these cortical areas are less interconnected or more differentiated from other brain regions, and conversely, increased regional MS. There was a significant negative correlation between the mean control regional MS and the *t* statistics for the case‐control differences in regional MS (Pearson's *r* = −0.82, *p* < 0.001; Figure 2D). This finding indicates that highly connected regions are more likely to show more significant differences in MS between cases and controls.

We next analyzed the correlation between PD‐related changes in MS and several clinical features, including MMSE, HAMD, HAMA, MoCA, and UPDRS Part III scores and Hoehn–Yahr stage. There were no significant correlations between MS values of significantly increased or decreased regions and clinical features (Supplemental Result S3 and Table S3 in Data S1).

### 3.3 Gene expression related to regional changes in MS

**FIGURE 3:**
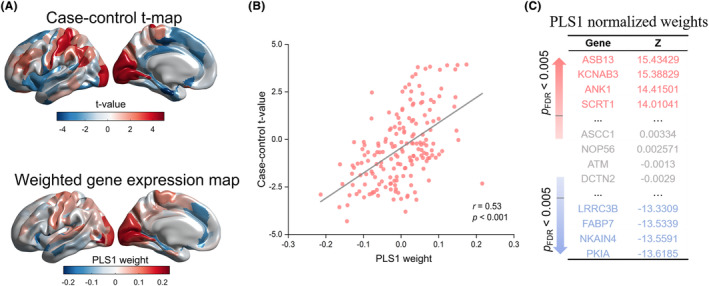
Gene expression profiles related to morphometric similarity differences. (A) Cortical maps of Case‐control regional MS differences and regional PLS1 gene expression weighted values in the left hemisphere. (B) Scatterplot of regional PLS1 weights (weighted sum of 10,027 gene expression scores) and regional MS differences demonstrating positive correlation (Pearson's *r* = 0.53, *p* < 0.001). (C) The PLS1 gene expression weights.

We used PLS regression to identify differences in gene expression that were correlated with the anatomical distribution of MS differences in the left hemisphere (Figure 3A). The first component of the PLS (PLS1) was a linear combination of the gene expression values most associated with regional variation in MS (permutation test, *p* < 0.001). In our study, PLS1 explained 28% of the variance (permutation test, *p* < 0.001), which was the component with the highest percentage of variance explained. Second, component 2 explained 15% of the variance. Of these components, only PLS1 explained a significant proportion of the variance in MS (permutation test, *p* < 0.001; Figure S2). Thus, we selected PLS1 for the next analysis. We observed that PLS1 gene expression weights were positively correlated with case–control *t* values, implying that positively (or negatively) weighted PLS1 genes were overexpressed in increased (or decreased) regional MS in PD patients compared to controls (Pearson's *r* = 0.53, *p* < 0.001; Figure 3B). Then, we identified a 1238 PLS1+ gene set (Z > 5) and a 784 PLS1− gene set (Z < −5) after normalizing PLS1 weights (all *p*_FDR_ < 0.05; Figure 3C). The PLS1 gene names and Z‐score weights were provided in Dataset S1.

We found 64 previously defined PD‐related genes from the AHBA dataset (https://human.brain‐map.org/microarray/search) after filtering by overlapping with 10,027 background genes. Then, we selected 13 overlapping genes from the known 64 PD‐related genes and 2022 PLS1 genes. Each of the 13 overlapping genes was correlated with case–control changes in MS in the left hemisphere (all *p*_FDR_ < 0.001; Figure S3). Of which, seven genes (MAPK1, SNCA, CASK, YWHAZ, MAPK3, YWHAH, and PSMB1) showed negative correlations with MS changes, while six genes (LRRK2, ATP1A3, FGR, SNCB, HSPA1L, and MPP1) showed positive correlations.

### 3.4 Transcriptional correlations between PD‐related changes in MS and differentially expressed genes in post‐mortem brains

We obtained a total of 43 overlapping genes from the PLS1− genes and the DEGs that were upregulated in the post‐mortem brain cortex of PD patients.^34^ Then, we found that the PLS1− gene weights were positively correlated with the upregulated differential gene expression (DGE) values (*r* = 0.37, *p* = 0.01; Figure S4), but not with downregulated DGE values in the brain cortex of PD patients (*r* = 0.10, *p* = 0.58). However, this specific positive correlation was unique to the DEGs identified in the cortex of PD patients and did not exist in the subcortical brain regions or for the other synucleinopathies, including dementia with Lewy bodies (DLB) and multiple system atrophy (MSA).^34^, ^35^ Additionally, PLS1+ gene weights were not significantly correlated with upregulated DGE values in the brain cortex (*r* = 0.08, *p* = 0.83), and not with upregulated DGE in the subcortical brain regions of PD patients (*r* = 0.207, *p* = 0.15). Similarly, PLS1+ gene weights were not significantly correlated with upregulated DGE values in the post‐mortem brain tissue of patients with DLB (*r* = −0.20, *p* = 0.47), or MSA (*r* = −0.06, *p* = 0.83).

### 3.5 Enrichment pathways of genes related to changes in MS

**FIGURE 4:**
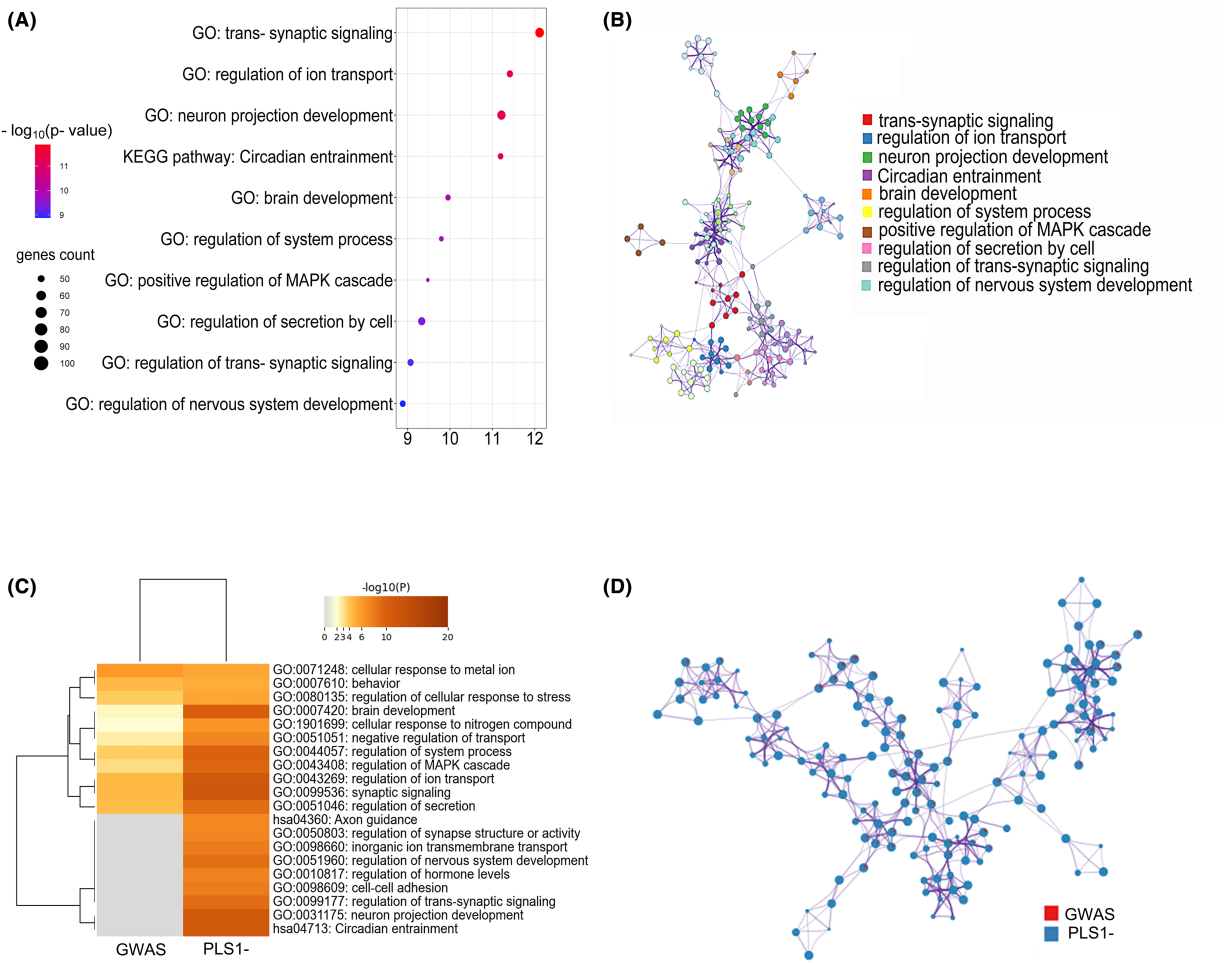
Enrichment analysis of genes transcriptionally related to morphometric similarity. (A) Bubble plot of the top 10 significant enrichment terms. PLS1− genes were significantly enriched in Gene Ontology (GO) biological processes and Kyoto Encyclopedia of Genes and Genomes (KEGG) pathways (*p* < 0.05). The color of the circle represents −log_10_ (*p*‐value), and the size of the circle represents the number of genes in the same term. (B) Visualization of enriched ontology terms. A circle node represents a given term, and its size depends on the number of genes in that term. Circle nodes of the same color belong to the same cluster. (C) Heatmap visualization of overlapped enrichment pathways between PLS1− genes and candidate risk genes from the genome‐wide association study (GWAS) of PD. The color of the cell represents −log_10_ (*p*‐value), and the white cell indicates the lack of that ontology term in the corresponding gene list. (D) Enrichment network visualization for results from the two gene lists, where nodes are represented by pie charts indicating their associations with each input study. Red is for genes from GWAS, and blue is for PLS1− genes.

We first analyzed the PLS1− gene set for significantly enriched Gene Ontology (GO) biological processes and Kyoto Encyclopedia of Genes and Genomes (KEGG) pathways. The enrichment analyses revealed that the PLS1− genes were mainly enriched in the GO biological processes, such as “trans‐synaptic signaling,” “regulation of ion transport,” “neuron projection development,” “brain development,” “positive regulation of MAPK cascade,” “regulation of trans‐synaptic signaling,” and “regulation of nervous system development,” and significantly enriched in one KEGG pathway, which was “circadian entrainment” (*p* < 0.05; Figure 4A,B). In addition, PLS+ genes were enriched in non‐neuron‐specific biological processes, including “metal ion transport,” “regulation of ion transport,” and “regulation of protein transport,” but not in KEGG pathways (*p* < 0.05; Supplemental Result S4 and Figure S5 in Data S1). To investigate the same enrichment pathways between PLS1− genes and polygenic risk genes for PD, we applied a multi‐gene‐list meta‐analysis between PLS1− genes and candidate risk genes from the genome‐wide association study (GWAS) of PD.^39^ This analysis indicated that PLS1− genes shared the top 10 same enrichment pathways with genes from the GWAS study, thus indicating that the PLS1− genes were functionally consistent with prior studies. The top 10 pathways included “synaptic signaling,” “regulation of ion transport,” “neuron projection development,” “regulation of system process,” “regulation of MAPK cascade,” “brain development,” “Circadian entrainment,” “regulation of secretion,” “regulation of trans‐synaptic signaling,” and “inorganic ion transmembrane transport” (*p* < 0.05; Figure 4C,D).

### 3.6 Cell‐type‐specificity of genes related to changes in MS

**FIGURE 5:**
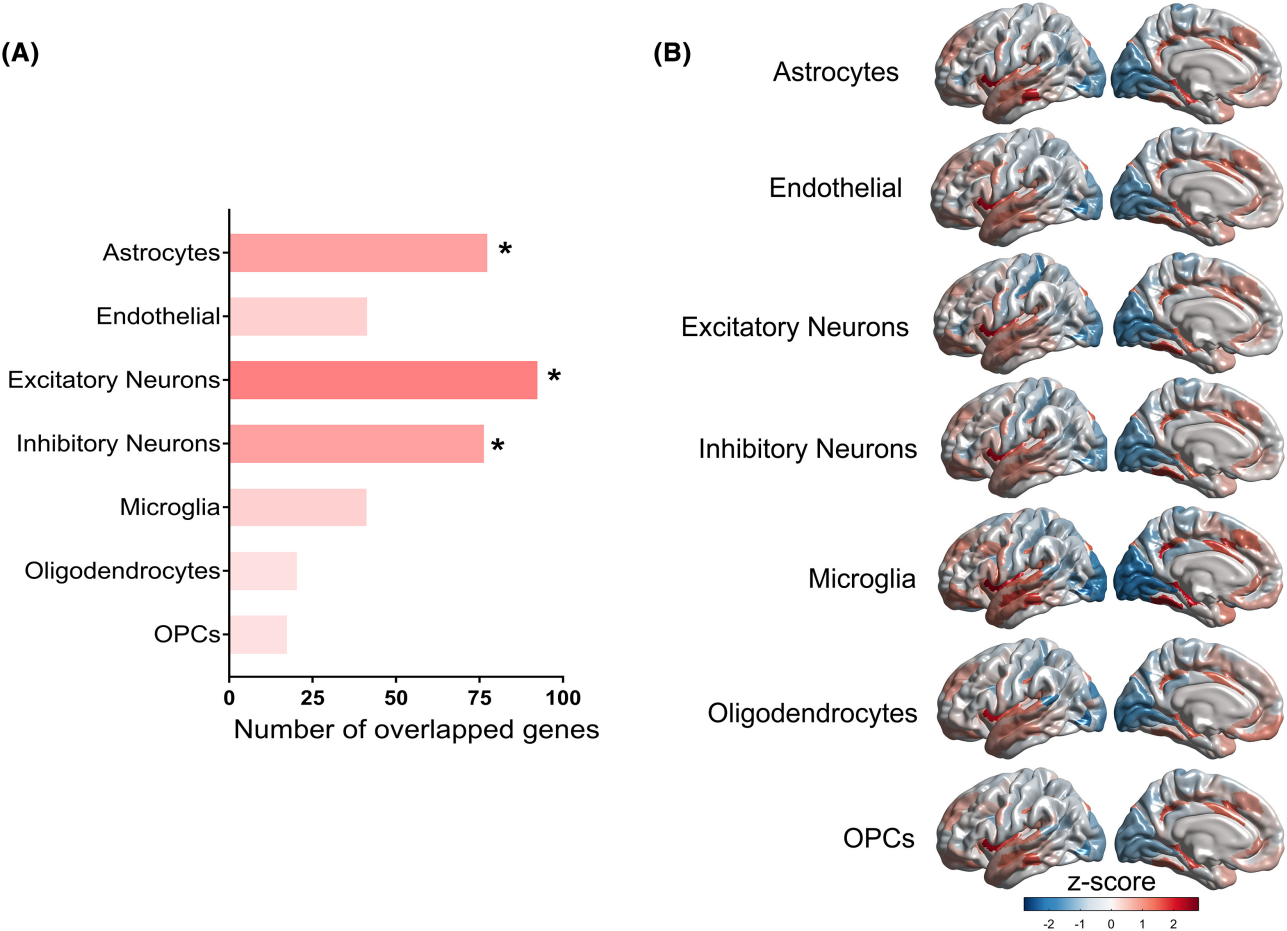
Cell type‐specific analysis of genes related to MS changes. (A) The number of overlapped genes for each cell type (Astrocytes: number = 78, *p* = 0.001; Excitatory neurons: number = 93, *p* < 0.001; Inhibitory neurons: number = 77, *p* = 0.004; Microglia: number = 42, *p* = 1.0; Endothelial: number = 42, adjusted *p* = 1.00; Oligodendrocyte precursors (OPCs): number = 18, *p* = 0.14; Oligodendrocytes: number = 21, *p* = 0.19; all *p* values were derived from permutation tests adjusted by FDR). An asterisk represents *p*_FDR_ < 0.05. (B) Regional gene expression maps of each cell type from overlapping genes between PLS1− genes and each cell type‐specific gene.

After assigning PLS1 genes to seven canonical cell classes,^23^, ^37^ we obtained specific cell types enriched for the regional MS changes in our study. In the PLS1− gene list, a total of 93 genes were significantly involved in excitatory neurons (*p* < 0.001, FDR‐corrected), 78 genes were significantly involved in astrocytes (*p* = 0.001, FDR‐corrected), and 77 genes were significantly involved in inhibitory neurons (*p* = 0.004, FDR‐corrected) (Figure 5). In the PLS1+ genes list, a total of 164 genes were significantly involved in excitatory neurons (*p* = 0.009, FDR‐corrected), and 109 genes were significantly involved in inhibitory neurons (*p* = 0.003, FDR‐corrected) (Figure S6).

## 4 DISCUSSION

Our study found that the cortical pattern of morphometric similarity differences in early drug‐naive PD patients was significantly associated with regional transcriptome signatures correlated with PD‐related genes. Furthermore, we obtained the PLS1− genes that spatially correlated with the PD‐related MS changes and were enriched in previously reported upregulated genes in the post‐mortem brain of PD patients, while also sharing the same enrichment pathways with genes from a GWAS study. In addition, those genes were involved in astrocytes and neuronal cells and functionally enriched in neuron‐specific biological processes related to trans‐synaptic signaling and nervous system development.

Recently, complex network theories have substantially contributed to our understanding of the structure and function of the brain as a large‐scale neural network, that is, connectome. Within this framework, pathology is considered a phenomenon of abnormal connections and interactions between distributed brain regions rather than the result of focal lesions.^12^ Unlike conventional morphometric networks, which construct intersubject networks using a specific morphological indicator of a group of subjects, the MS network reflects the inter‐regional morphological similarity information from multiple MRI anatomical indices in individual subjects. Morphological similarity mapping provided a robust and biologically credible cortical pattern of difference in patients with neuropsychiatric disorders.^18^ This study showed that MS was mainly decreased in cingulate, frontal, and temporal cortical areas and increased in parietal and occipital cortical areas in early drug‐naive PD patients. Prior studies suggested that more morphometrically similar cortical regions implied those regions have similar cytoarchitectonic classes and are more likely to be connected anatomically.^19^, ^21^ Based on this theory, we proposed that the reduced MS in cingulate, frontal, and temporal cortices in PD patients indicated greater architectonic differentiation and decreased axonal connectivity between these regions and the rest of the cortex. Consistent with our findings, previous DTI studies of early‐stage PD revealed decreased white matter connectivity in several cortico‐striatal structural networks, including putamen‐temporal, caudate‐frontal, and caudate‐insula connectivity.^40^ A multimodal imaging study showed that parieto‐occipital regions had higher spatial convergence and higher interregional correlations between posterior cortical regions.^41^ Additionally, the increased functional connectivity along fronto‐parietal connections and in the posterior default mode network was negatively associated with cognitive function in PD patients.^41^, ^42^ The increased MS may be related to compensatory reallocation and a maladaptive response to neurodegenerative processes in early drug‐naive PD patients.

Given the complex pathogenesis of PD, the PD‐related MS changes may appear to be shaped by various factors, including genetic, neuronal, molecular, and cellular variations.^43^, ^44^ Using PLS regression to spatially link neuroimaging phenotypes to gene expression signatures from the AHBA dataset, we identified a set of genes with significantly positive/negative weights on PLS1 that are associated with the MS changes in PD patients. Of the PD‐related genes, SNCA was negatively associated with the MS changes in PD patients. Pathologically, the abnormal aggregation of Lewy bodies, mainly consisting of α‐synuclein (encoded by SNCA), is the hallmark of PD.^45^ It was reported that aggregated α‐synuclein proteins caused by causative point SNCA variants and the overexpression of normal SNCA could accelerate dopaminergic neuron neurodegeneration through multiple pathological processes.^46^ In addition, the discovered MAPK1 gene showed the strongest negative association, which is recognized as an important effector of neuronal apoptosis that leads to neuronal death in PD.^47^, ^48^ Moreover, MAPK1, as a functional target of microRNA‐125b‐5p, could exert neuroprotective effects against cytotoxicity by attenuating its overexpression in both human and mouse PD cell models.^49^ We further compared PLS1− genes with DEGs from post‐mortem brains with synucleinopathies, including PD, DLB, and MSA.^34^, ^35^ In our study, the specific relationship between the PLS1− genes overexpressed in the cingulate, frontal, and temporal cortical areas of decreased MS and the PD‐related genes upregulated in the post‐mortem cortex confirmed the specificity of gene expression in different diseases and spatial distribution.^50^ This finding also provided further evidence that differences in regional gene transcription might shape spatial connectome dysfunction by driving brain areas selective vulnerability to neurodegenerative pathology.

Additional investigation indicated that PLS1− genes were significantly involved in astrocytes, excitatory, and inhibitory neurons and functionally enriched in neuron‐specific biological processes related to trans‐synaptic signaling and nervous system development. Astrocytes, known as the most numerous of the glial cells,^51^ participate in key processes related to CNS development and function, including neurotransmitter recycling, synaptic transmission regulation, and ionic balance.^52^, ^53^, ^54^ In addition to glutamate‐mediated excitotoxicity, astrocytes conduce to PD pathology by modulating Ca^2+^ and K^+^ homeostasis, which regulates dopaminergic neuron death.^55^, ^56^ Similarly, we found that excitatory and inhibitory neurons were closely associated with the abnormal gene expression driving the decreased MS in PD. Previous studies suggested that excitatory/inhibitory (E/I) balance effectively sustains homeostasis in the CNS and that an E/I imbalance consisting of various neurotransmitters, such as DA, glutamate, GABA, acetylcholine, and serotonin, is involved in the pathological progression of PD.^57^, ^58^ The progressive DA decrease in the substantia nigra pars compacta (SNc), which diminishes the excitability of GABAergic interneurons and concurrently impairs SNc neurotransmission to the dorsal striatum, which contributes to the pathophysiology of PD.^59^ In recent years, developing therapeutics that target the GABAergic system in addition to the dopaminergic system may become a new strategy for the treatment of PD.^60^

There were several limitations to our study. First, although we determined the PLS1− gene set that spatially correlated with the PD‐related MS changes and then verified the specificity of those genes based on previously upregulated genes in post‐mortem brain samples from PD patients, we were unable to ensure that every transcriptionally dysregulated gene in the PLS1− gene set contributed to morphological abnormalities. Further in vitro and in vivo experiments are essential to validate our findings. Second, this study used the public AHBA gene data measured in post‐mortem brains taken from six donors without any history of neurological disorders. The gene expression profiles based on “normal” brain tissue may limit the examination of the relationship between transcriptional data and macroscale MS abnormalities across groups, with possible individual patient effects being out of scope. In addition, only the left hemisphere data were applied in our study, as the AHBA dataset included only two available right hemisphere data. Therefore, the gene expression related to regional changes in MS could not reflect the transcriptional information of the whole brain. In the future, collecting more samples from the whole brain of post‐mortem PD patients will be important to determine the differences between hemispheres and advance the understanding of regional selective vulnerability in PD. Additionally, individual differences in human brain structure, function, and behavior can be attributed to several factors, including genetic variations, environmental exposure, and their interactions. In addition to genetic factors, environmental factors are crucial for neuropsychiatric disorders. Although our study has discovered the transcriptional signatures related to brain morphological phenotypes, the environmental exposure related to these phenotypes remains unclear.^61^ Finally, although this cross‐sectional study has shown the specific gene expression pattern spatially related to regional MS changes in early‐stage PD, longitudinal studies will be of great value in exploring the pattern of MS changes in PD patients with disease progression.

In summary, our study found differences in cortical patterns in PD, indicating that MS was mainly decreased in the cingulate, frontal, and temporal cortical areas. When combing spatial morphometric information with transcriptional signatures, we discovered that MS‐related genes were involved in astrocytes, excitatory, and inhibitory neurons and were functionally enriched in neuron‐specific biological processes related to trans‐synaptic signaling and nervous system development. These findings have advanced our understanding of the microscale genetic and cellular mechanisms driving macroscale morphological abnormalities and have provided potential targets for future therapeutic trials.

## AUTHOR CONTRIBUTIONS

WL and YZ organized the project and critically revised the manuscript. YW and YX analyzed the transcriptional data. YW drafted the preliminary manuscript. YX and MY collected data and performed statistical analysis. XW and JR critiqued the statistical analysis. All authors contributed to the article and approved the submitted manuscript.

## FUNDING INFORMATION

This work was supported by the National Natural Science Foundation of China (NSFC) (No. 81571348, 81701675, 81903589, 81701671), the National Key Research and Development Program of China (2017YFC1310300, 2017YFC1310302, and 2016YFC1306600), the Jiangsu Provincial Natural Science Foundation of China (BK20151077), the Medical Science and technology development Foundation, Nanjing Department of Health (No. JQX21006).

## CONFLICT OF INTEREST STATEMENT

The authors have no conflict of interest to report.

## CODE AVAILABILITY STATEMENT

The code for gene expression analysis is openly available at https://github.com/BMHLab/AHBAprocessing. The codes for MSN analysis and PLS can be found at https://github.com/SarahMorgan/Morphometric_Similarity_SZ. The code for permutation testing is openly available at https://github.com/frantisekvasa/rotate_parxellation.

## Supporting information


Data S1.

Dataset S1.

## Data Availability

The data that support the findings of this study are available from the corresponding author upon reasonable request.
