# Supplementary material for: Morphometric similarity differences in drug‐naive Parkinson's disease correlate with transcriptomic signatures
Source: CNS Neurosci Ther. 2024 Mar 26;30(3):e14680. doi: 10.1111/cns.14680 (PMC10964038; doi:10.1111/cns.14680)
Supplement: Supplementary file 1 — Data S1. [file CNS-30-e14680-s002.zip › Supplemental Result.docx]

**Supporting information for**

**“Morphometric Similarity Differences in Drug-naive Parkinson’s Disease Correlate with Transcriptomic Signatures”**

**Yajie wang^1,2#^, Yiwen Xiao^3#^, Yi Xing****^1^, Miao Yu^1^, Xiao Wang^4^, Jingru Ren^1^, Weiguo Liu^1*^, Yuan Zhong^3*^**

^1^ Department of Neurology, The Affiliated Brain Hospital of Nanjing Medical University, Nanjing, 210029, China

^2^ Department of Neurology, the First People’s Hospital of Yancheng, Yancheng, 224000, China

^3^ School of Psychology, Nanjing Normal University, Nanjing, 210097, Jiangsu, China

^4^ Department of Radiology, The Affiliated Brain Hospital of Nanjing Medical University, Nanjing, 210029, China

**^*^ Corresponding Author**

Weiguo Liu, Department of Neurology, The Affiliated Brain Hospital of Nanjing Medical University, Nanjing 210029, China; ORCID: <https://orcid.org/0000-0001-5916-9837>; Email: [wgliunbh@sina.com](mailto:wgliunbh@sina.com)

Yuan Zhong, School of Psychology, Nanjing Normal University, Nanjing 210097, Jiangsu, China; Email: [zhongyuan@njnu.edu.cn](mailto:zhongyuan@njnu.edu.cn)

^#^ These authors have contributed equally to this work

**Relevant conflicts of interest/financial disclosures related to the manuscript:** The authors have no conflict of interest to report.

**Funding:** This work was supported by the National Natural Science Foundation of China (NSFC) (No. 81571348, 81701675, 81903589, 81701671), the National Key Research and Development Program of China (2017YFC1310300, 2017YFC1310302, and 2016YFC1306600), the Jiangsu Provincial Natural Science Foundation of China (BK20151077).

**Supplemental Result**

**1. Quality control**

The data of 170 PD patients and 123 HCs were finally analyzed after excluding 10 patients and 9 HCs due to excessive head motion (cumulative translation or rotation > 3.0 mm or 3.0°, n = 1). The demographic and clinical characteristics of all subjects were summarized in **Table S1**. To check for differences in motion and image quality between the two groups, we calculated the Euler number for each T1w image. This approach was proposed by Rosen et al.[3] as a way to quantitatively assess image quality[1,2]. There was no significant difference in the Euler number between the two groups (*t* = 0.10, *p* = 0.96; **Fig. S1**).

**Table S1 Clinical and Demographic Characteristics**

|  | PD  (n=170) | HC  (n=123) | P-value |
| --- | --- | --- | --- |
| Age, years | 58.46±8.64 | 58.66±5.92 | 0.815^a^ |
| Gender, male (%) | 78 (45.9%) | 54 (43.9%) | 0.737^b^ |
| Education, years | 9.65±4.11 | 11.18±4.13 | 0.001^c^ |
| Disease duration, years | 2.16±2.30 | / | / |
| MMSE score | 26.89±3.34 | 28.69±2.21 | < 0.001^c^ |
| MoCA score | 21.76±4.83 | / | / |
| HAMD score | 9.77±6.92 | 2.51±3.41 | < 0.001^c^ |
| HAMA score | 6.97±5.42 | / | / |
| UPDRS part III | 21.66±10.81 | / | / |
| H-Y stage | 1.61±0.52 | / | / |

Data were expressed as mean ± SD or n (%).

^a^ Two-sample t-test (two-sided). ^b^ Chi-square test. ^c^ Mann-Whitney U-test (two-sided).

Abbreviations: PD, Parkinson’s disease; HC, healthy control; MMSE, The Mini Mental State Examination; MoCA, The Montreal Cognitive Assessment; HAMD, The Hamilton Depression Rating Scale; HAMA, The Hamilton Anxiety Scale; UPDRS, Unified Parkinson’s Disease Rating Scale; H-Y, Hoehn and Yahr.


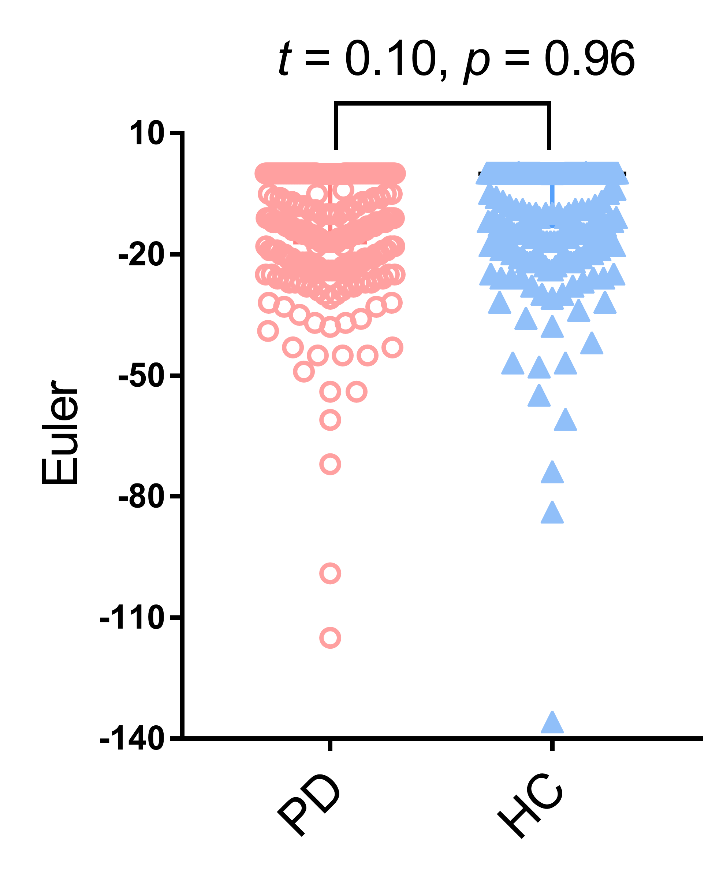


**Fig. S1 The comparison of the Euler number between the PD and HC groups.** There was no significant difference in the Euler number between the two groups (PD: -20.91±14.61, n = 170; HC: -21.01±16.27, n = 123; two-sample t-test: *t* = 0.10, *p* = 0.96).

**2. Regional MS differences between in PD patients and controls**

Compared to HC group, the PD group showed significant decreased/increased regional MS weights in 31 brain regions (all *p* < 0.05, FDR-corrected). We listed the names of brain areas and their corresponding regions anatomical labels, mean MS value and t-statistic values in **Table S2**.

**Table S2 Regions showing abnormal MS in PD patients**

| Area ID | Area name | Hemisphere | Primary section | PD MS | Control MS | t-statistic | Cohen’s d |
| --- | --- | --- | --- | --- | --- | --- | --- |
| 1 | V1 | L | primary Visual Cortex | -0.025 | -0.074 | 3.94 | 0.494 |
| 1 | V1 | R | primary Visual Cortex | -0.026 | -0.074 | 3.83 | 0.481 |
| 2 | V2 | L | Early Visual Cortex | -0.023 | -0.072 | 3.91 | 0.493 |
| 4 | V2 | R | Early Visual Cortex | -0.024 | -0.072 | 3.86 | 0.488 |
| 5 | V3 | L | Early Visual Cortex | -0.015 | -0.066 | 3.68 | 0.475 |
| 5 | V3 | R | Early Visual Cortex | -0.016 | -0.067 | 3.68 | 0.476 |
| 6 | V4 | L | Early Visual Cortex | -0.004 | -0.06 | 3.82 | 0.504 |
| 9 | 3b | L | Somatosensory cortex | -0.023 | -0.071 | 3.9 | 0.486 |
| 9 | 3b | R | Somatosensory cortex | -0.02 | -0.071 | 4.04 | 0.502 |
| 40 | 24dd | L | Mid Cingulate Cortex | 0.012 | 0.067 | -3.8 | -0.438 |
| 51 | 1 | L | Somatosensory cortex | -0.007 | -0.054 | 3.66 | 0.456 |
| 51 | 1 | R | Somatosensory cortex | -0.003 | -0.053 | 3.73 | 0.456 |
| 52 | 2 | L | Somatosensory cortex | -0.013 | -0.06 | 3.8 | 0.465 |
| 73 | 8C | R | Dorsolateral Prefrontal Cortex | 0.024 | -0.018 | 3.71 | 0.389 |
| 86 | 9-46d | R | Dorsolateral Prefrontal Cortex | 0.01 | -0.039 | 3.43 | 0.423 |
| 99 | 43 | R | Posterior Opercular Cortex | 0.064 | 0.113 | -3.39 | -0.43 |
| 100 | OP4 | R | Posterior Opercular Cortex | 0.074 | 0.123 | -3.66 | -0.457 |
| 106 | PoI2 | R | Insular and Frontal Opercular Cortex | 0.041 | 0.095 | -3.45 | -0.479 |
| 109 | MI | R | Insular and Frontal Opercular Cortex | 0.013 | 0.079 | -4.04 | -0.513 |
| 110 | Pir | L | Insular and Frontal Opercular Cortex | 0.005 | 0.048 | -3.56 | -0.425 |
| 110 | Pir | R | Insular and Frontal Opercular Cortex | -0.004 | 0.047 | -3.83 | -0.47 |
| 111 | AVI | R | Insular and Frontal Opercular Cortex | 0.026 | 0.079 | -3.53 | -0.445 |
| 115 | FOP2 | R | Insular and Frontal Opercular Cortex | 0.032 | 0.084 | -3.64 | -0.461 |
| 117 | AIP | L | Superior Parietal Cortex | 0.012 | -0.041 | 3.72 | 0.474 |
| 118 | EC | L | Medial Temporal Cortex | 0.008 | 0.068 | -4.31 | -0.535 |
| 149 | PFm | L | Inferior Parietal Cortex | -0.002 | -0.042 | 3.3 | 0.397 |
| 150 | PGi | L | Inferior Parietal Cortex | 0.002 | -0.044 | 3.43 | 0.449 |
| 164 | 25 | L | Anterior Cingulate and Medial Prefrontal Cortex | 0.033 | 0.084 | -3.46 | -0.453 |
| 172 | TGv | R | Lateral Temporal Cortex | 0.04 | 0.093 | -3.56 | -0.456 |
| 179 | a32pr | R | Anterior Cingulate and Medial Prefrontal Cortex | 0.027 | 0.08 | -3.43 | -0.422 |
| 180 | p24 | R | Anterior Cingulate and Medial Prefrontal Cortex | 0.047 | 0.097 | -3.32 | -0.444 |

The table showed the names of 31 statistically significant brain areas and their corresponding regions anatomical labels, mean MS value in PD patients and controls, t-statistic (all *p* < 0.05 after FDR-corrected).

**3. Correlation between MS values and clinical features**

To explore the clinical significance of the morphometric differences, we analyzed the relationship between the MS values and clinical assessments including Unified Parkinson’s Disease Rating Scale (UPDRS part III), Hoehn and Yahr (H-Y) stage, Hamilton Depression Rating Scale (HAMD), Hamilton Anxiety Scale (HAMA), Mini Mental State Examination (MMSE) and Montreal Cognitive Assessment (MoCA). For each patient, we added up the MS values of each statistically significant brain area belonging to the same region after Z-normalization, and then calculated the correlation between the sum MS values of each region and clinical features. The results of the Pearson’s correlation analysis were list in **Table S3**.

Pearson’s correlation analysis showed that the MS values that were decreased in brain regions correlated negatively with MoCA scores, whereas the MS values that were increased in brain regions correlated positively with MoCA scores in PD patients (all *p* < 0.05). However, there was no significant correlation between the MS values and the other clinical features.

**Table S3 Correlation analysis between MS values of significant abnormal regions and clinical features in PD patients**

|  | Brain regions | MMSE | | HAMD | | HAMA | | MoCA | | UPDRS Ⅲ | | H-Y | |
| --- | --- | --- | --- | --- | --- | --- | --- | --- | --- | --- | --- | --- | --- |
|  |  | r | p | r | p | r | p | r | p | r | p | r | p |
| Decreased MS |  |  |  |  |  |  |  |  |  |  |  |  |  |
|  | L_Mid Cingulate Cortex | 0.059 | 0.446 | 0.110 | 0.156 | 0.049 | 0.526 | 0.126 | 0.103 | -0.060 | 0.437 | -0.028 | 0.715 |
|  | R_Posterior Opercular Cortex | 0.008 | 0.916 | 0.059 | 0.446 | 0.076 | 0.324 | -0.022 | 0.779 | 0.022 | 0.777 | -0.020 | 0.795 |
|  | L_Insular and Frontal Opercular Cortex | -0.024 | 0.757 | 0.011 | 0.887 | 0.018 | 0.815 | -0.191 | 0.013 | 0.064 | 0.407 | 0.043 | 0.573 |
|  | R_Insular and Frontal Opercular Cortex | 0.076 | 0.328 | 0.022 | 0.775 | 0.021 | 0.790 | -0.035 | 0.653 | 0.000 | 0.997 | -0.005 | 0.943 |
|  | L_Medial Temporal Cortex | -0.006 | 0.934 | -0.057 | 0.463 | -0.071 | 0.358 | -0.090 | 0.245 | 0.028 | 0.717 | 0.040 | 0.606 |
|  | L_Anterior Cingulate and Medial Prefrontal Cortex | 0.090 | 0.244 | 0.016 | 0.834 | -0.020 | 0.797 | -0.094 | 0.222 | 0.018 | 0.817 | 0.053 | 0.496 |
|  | R_Lateral Temporal Cortex | 0.037 | 0.634 | 0.040 | 0.602 | -0.027 | 0.725 | -0.017 | 0.831 | -0.024 | 0.756 | -0.058 | 0.453 |
|  | R_Anterior Cingulate and Medial Prefrontal Cortex | -0.025 | 0.751 | 0.065 | 0.400 | 0.039 | 0.616 | -0.067 | 0.388 | 0.007 | 0.924 | 0.010 | 0.895 |
| Increased MS | L_primary Visual Cortex | 0.033 | 0.665 | 0.004 | 0.960 | 0.010 | 0.900 | 0.196 | 0.011 | -0.056 | 0.465 | -0.026 | 0.736 |
|  | R_primary Visual Cortex | 0.039 | 0.611 | 0.001 | 0.986 | 0.008 | 0.918 | 0.200 | 0.009 | -0.060 | 0.435 | -0.029 | 0.704 |
|  | L_early Visual Cortex | -0.021 | 0.783 | 0.034 | 0.658 | 0.045 | 0.557 | 0.134 | 0.083 | -0.015 | 0.850 | -0.004 | 0.960 |
|  | R_early Visual Cortex | -0.014 | 0.858 | 0.017 | 0.826 | 0.032 | 0.683 | 0.140 | 0.070 | -0.025 | 0.741 | -0.009 | 0.908 |
|  | L_somatosensory cortex | 0.032 | 0.676 | 0.038 | 0.628 | 0.029 | 0.708 | 0.197 | 0.010 | -0.043 | 0.580 | -0.017 | 0.824 |
|  | R_somatosensory cortex | -0.002 | 0.976 | 0.064 | 0.411 | 0.030 | 0.697 | 0.162 | 0.035 | -0.024 | 0.752 | -0.017 | 0.827 |
|  | R_DorsoLateral Prefrontal Cortex | -0.009 | 0.906 | 0.051 | 0.514 | 0.047 | 0.547 | 0.151 | 0.050 | -0.015 | 0.846 | -0.015 | 0.847 |
|  | L_Superior Parietal Cortex | -0.006 | 0.939 | 0.095 | 0.218 | 0.047 | 0.546 | 0.128 | 0.096 | 0.068 | 0.375 | 0.046 | 0.551 |
|  | L_Inferior Parietal Cortex | 0.050 | 0.518 | 0.037 | 0.635 | -0.001 | 0.992 | 0.212 | 0.006 | -0.038 | 0.622 | -0.004 | 0.962 |

All *r* values were obtained by Pearson’s correlation analysis. All *p* values were not survived after FDR-corrected, and were determined based on two-sided tests.

**4. Enrichment analysis of significantly positively weighted genes.**

We obtained 1238 PLS1+ gene set (Z > 5) after normalizing PLS1 weights. The enrichment analyses revealed that the PLS+ genes were mainly enriched in non-neuron-specific biological process, including “metal ion transport”, “regulation of ion transport”, “regulation of protein transport”, but no KEGG pathways (*p* < 0.05). The visualization of those significant enrichment pathways was shown in **Fig. S5**.


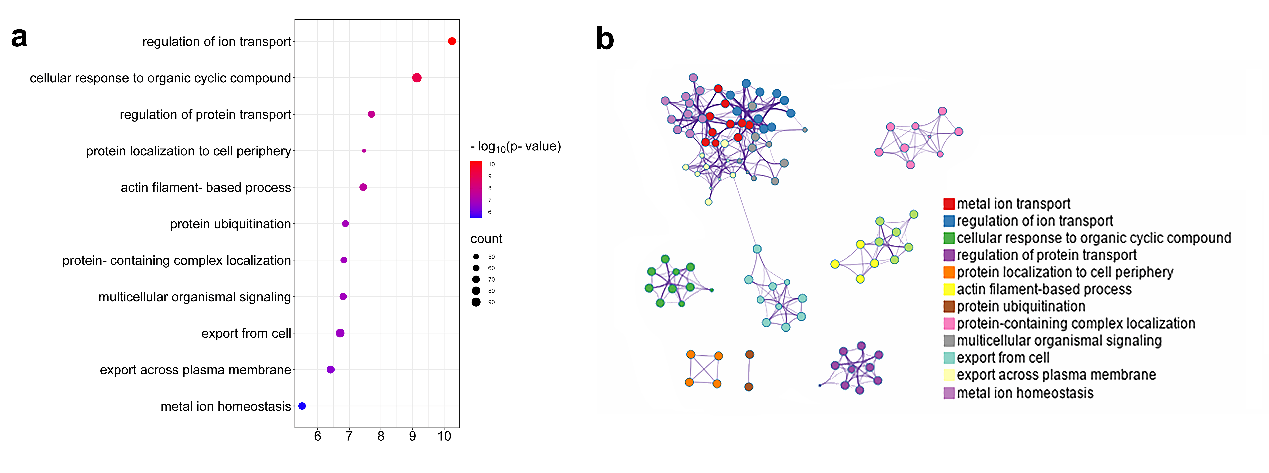


**Fig. S5 Enrichment pathways of PLS1+ gene list. (a)** Bubble plot of significant enrichment terms. The color of the circle represents -log10 (p-value), and the size of circle represents the number of genes in the same term. **(b)** Visualization of enriched ontology terms. A circle node represents a given term, and its size depends on the number of genes in that term. Circle nodes of the same color belong to the same cluster.

**Supplemental figures**

**Fig. S2**


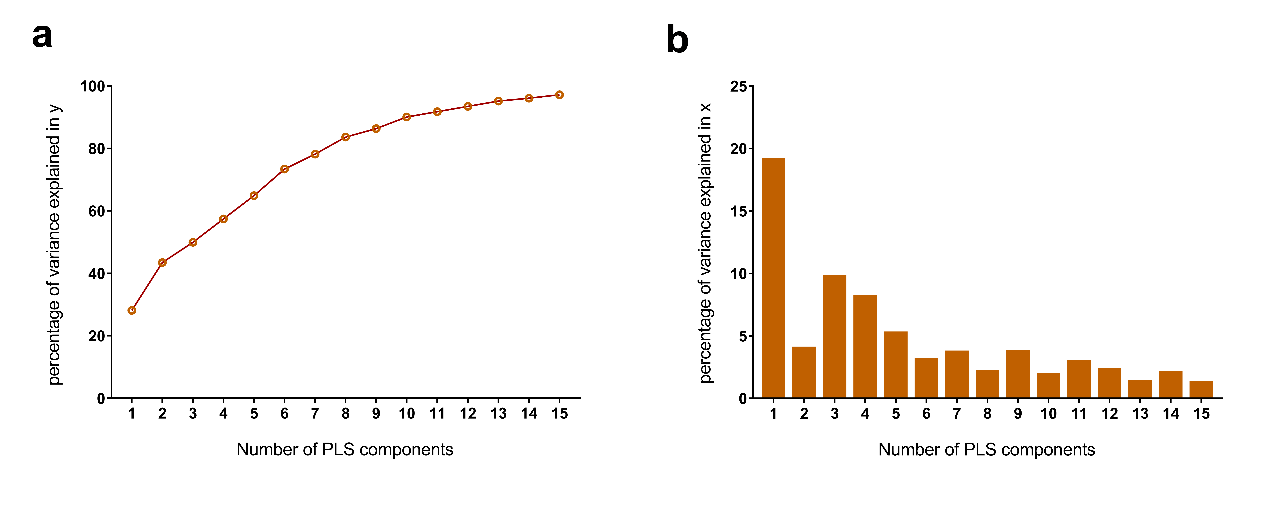


**Fig. S2 Percentage of variance explained by the top 15 PLS components. (a)** Percentage of variance explained in y (MS) by the top 15 PLS components. **(b)** Percentage of variance explained in x (gene expression) by the top 15 PLS components.

**Fig. S3**

**
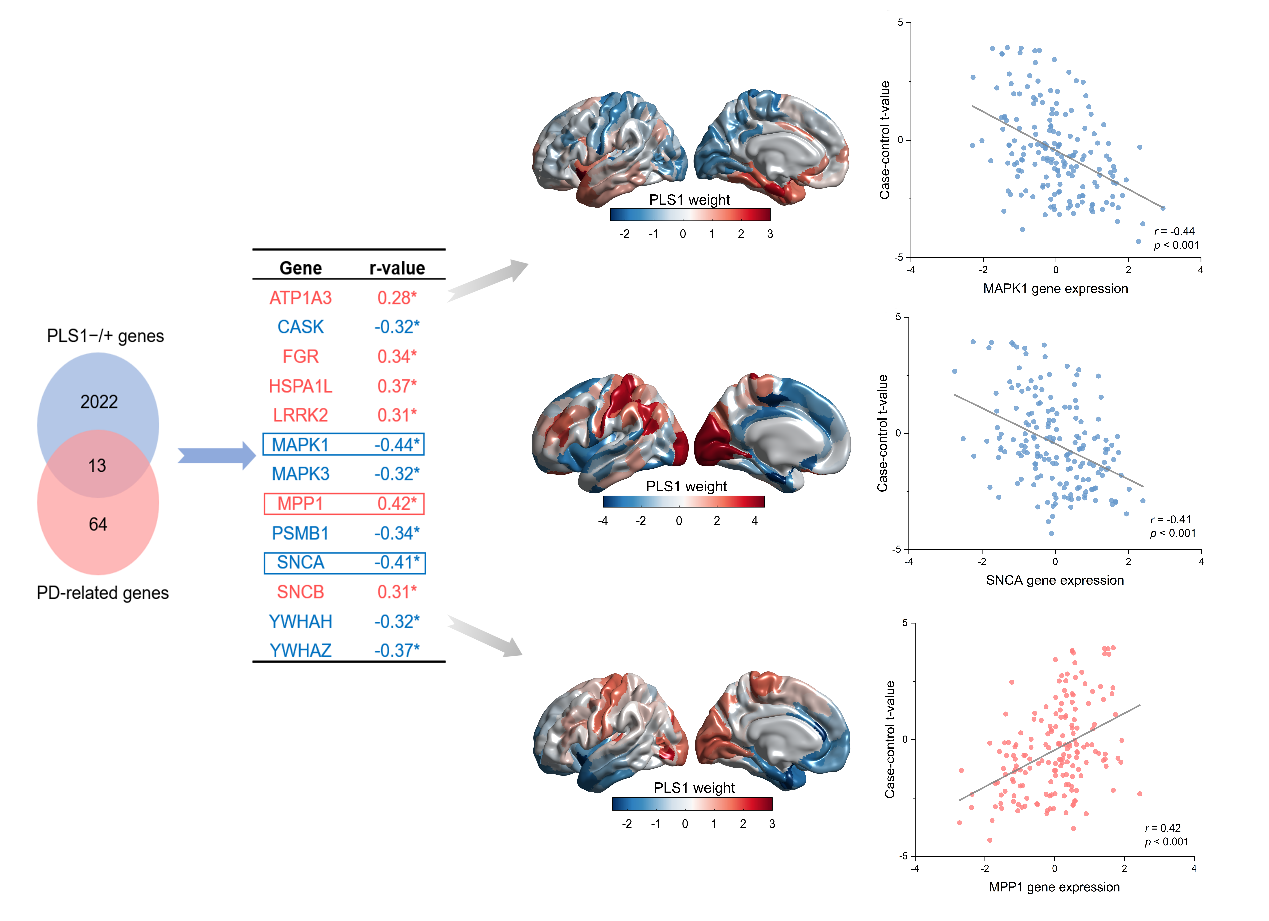
Fig. S3 Relationships between prior PD-related gene expression and regional MS differences.** 13 prior PD-related genes from AHBA dataset were all correlated with regional MS changes in the left hemisphere (all *p*_FDR_ < 0.001). In which, genes that were strongly positively weighted on PLS1 (e.g., MPP1) correlated positively with case–control MS differences (Pearson’s *r* = 0.42, *p* < 0.001), while genes that were strongly negatively weighted on PLS1 (e.g., MAPK1, SNCA) correlated negatively with case–control MS differences (MAPK1: Pearson’s *r* = -0.44, *p* < 0.001; SNCA: Pearson’s *r* = -0.41, *p* < 0.001).

**Fig. S4**


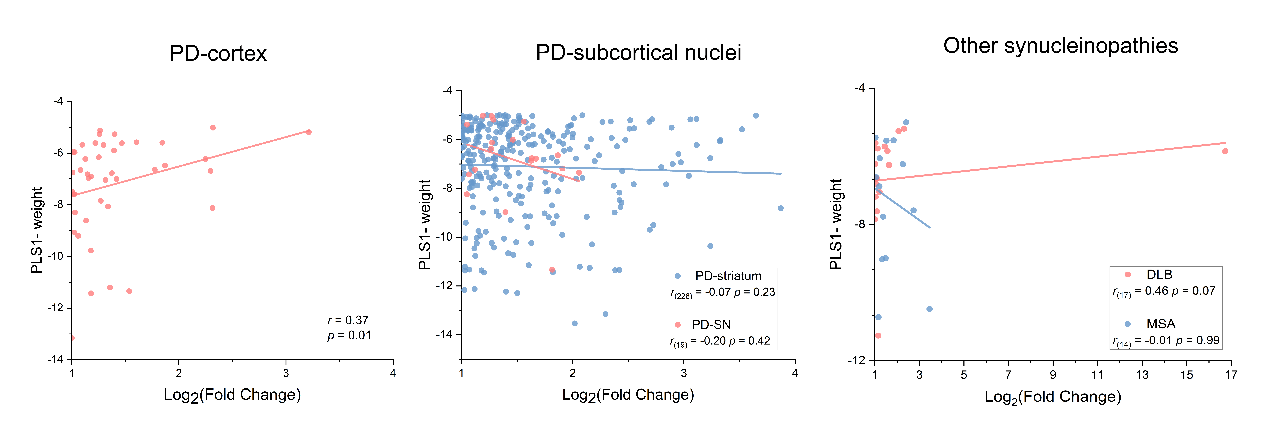


**Fig. S4 Associations between the PLS1− weighted gene expression with differential gene expression data from PD and others synucleinopathies in post-mortem brain tissues.** The PLS1− genes were significantly associated with the upregulated genes in cortex of PD (Spearman’s *r* = 0.37, *p* = 0.01), but not with the upregulated or downregulated genes in subcortical nuclei of PD (striatum: Spearman’s *r* = -0.07, *p* = 0.23; SN: Spearman’s *r* = -0.02, *p* = 0.42), as well as the other synucleinopathies (DLB: Spearman’s *r* = 0.46, *p* = 0.07; MSA: Spearman’s *r* = 0.01, *p* = 0.99).

Abbreviations: SN, substantia nigra; DLB, dementia with Lewy bodies; MSA, multiple system atrophy.

**Fig. S6**


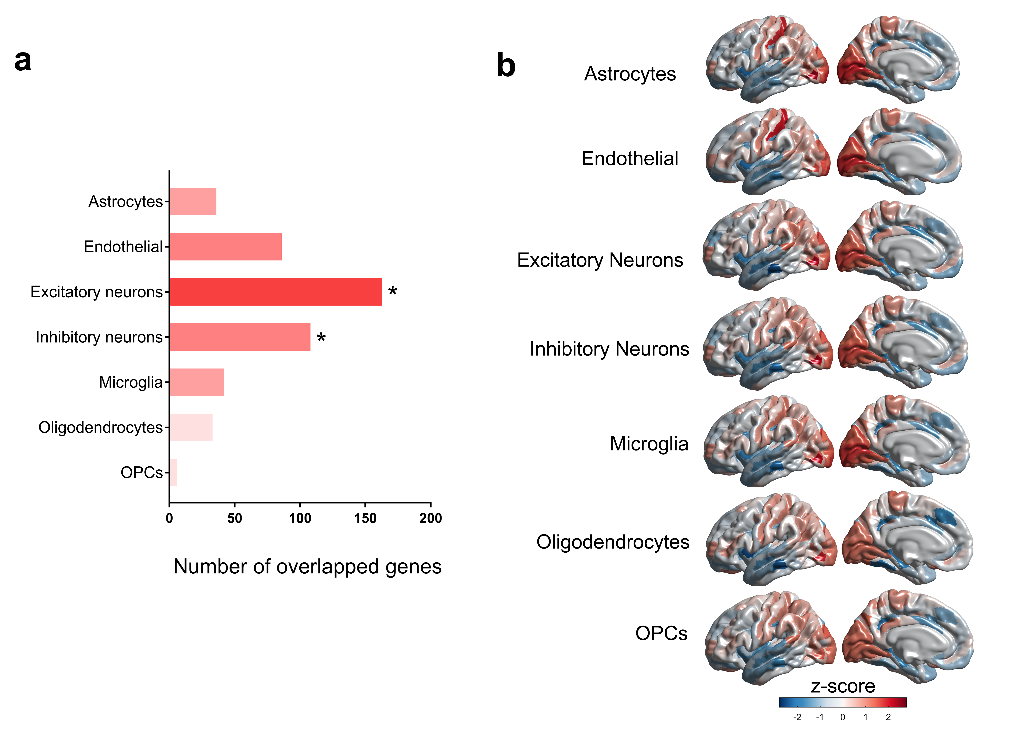


**Fig. S6 Cell type-specific analysis of genes related to MS changes. (a)** The number of overlapped genes for each cell type (Excitatory neurons: number = 164, *p* = 0.009; Inhibitory neurons: number = 109; all *p* values were derived from permutation tests adjusted by FDR). An asterisk represents *p*_FDR_< 0.05. **(b)** Regional gene expression maps of each cell type from overlapping genes between PLS1+ genes and each cell type-specific genes.

**References:**

1. Li J, Seidlitz J, Suckling J, et al. (2021). Cortical structural differences in major depressive disorder correlate with cell type-specific transcriptional signatures. Nat Commun*,* 12(1), 1647. doi:10.1038/s41467-021-21943-5

2. Morgan SE, Seidlitz J, Whitaker KJ, et al. (2019). Cortical patterning of abnormal morphometric similarity in psychosis is associated with brain expression of schizophrenia-related genes. Proc Natl Acad Sci U S A*,* 116(19), 9604-9609. doi:10.1073/pnas.1820754116

3. Rosen AFG, Roalf DR, Ruparel K, et al. (2018). Quantitative assessment of structural image quality. Neuroimage*,* 169, 407-418. doi:10.1016/j.neuroimage.2017.12.059
