# Supplementary figures and images for: Morphometric similarity differences in drug‐naive Parkinson's disease correlate with transcriptomic signatures
Source: CNS Neurosci Ther. 2024 Mar 26;30(3):e14680. doi: 10.1111/cns.14680 (PMC10964038; doi:10.1111/cns.14680)

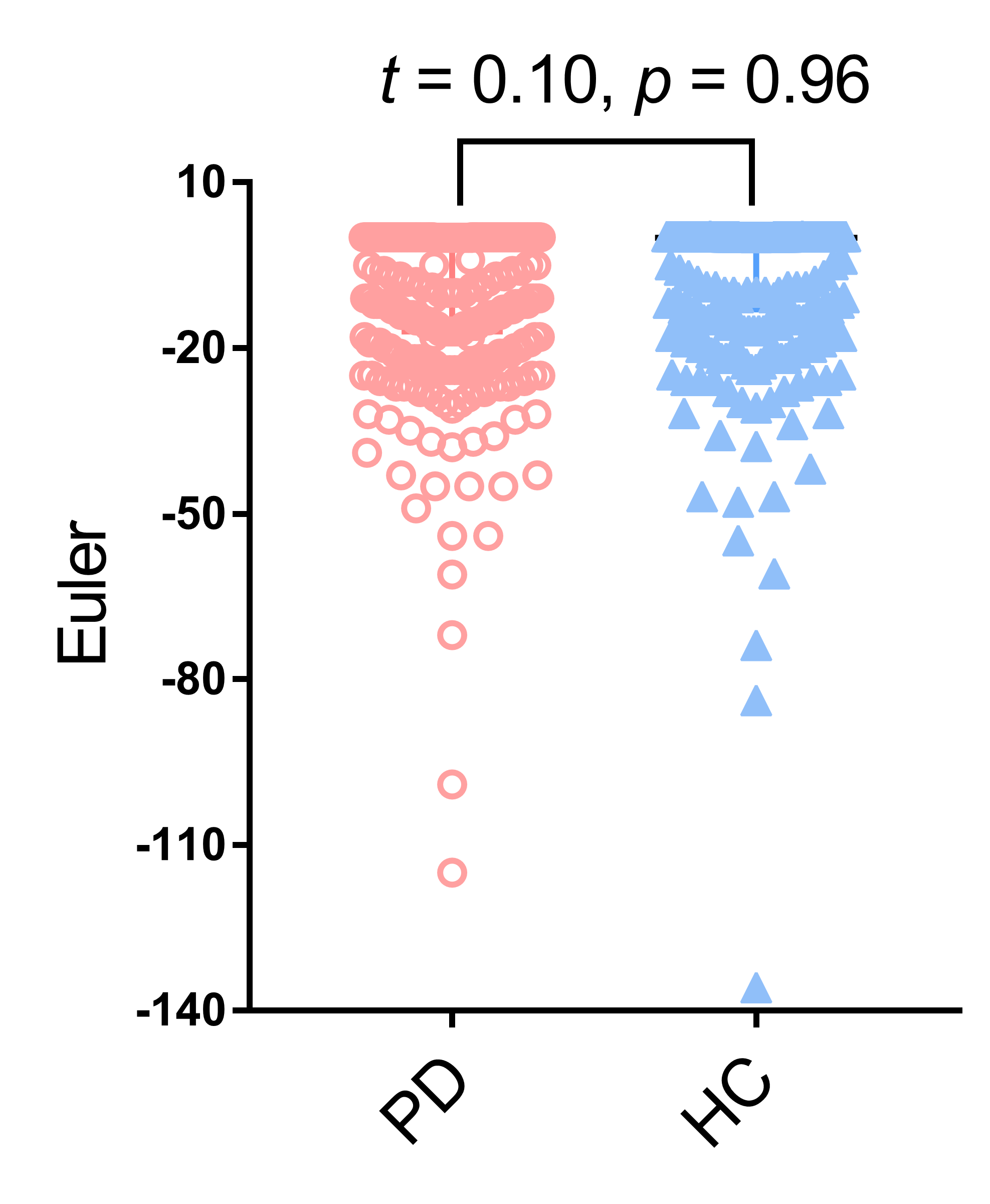

Supplement: Supplementary file 1 — Data S1. [file CNS-30-e14680-s002.zip › Fig. S1.tif]

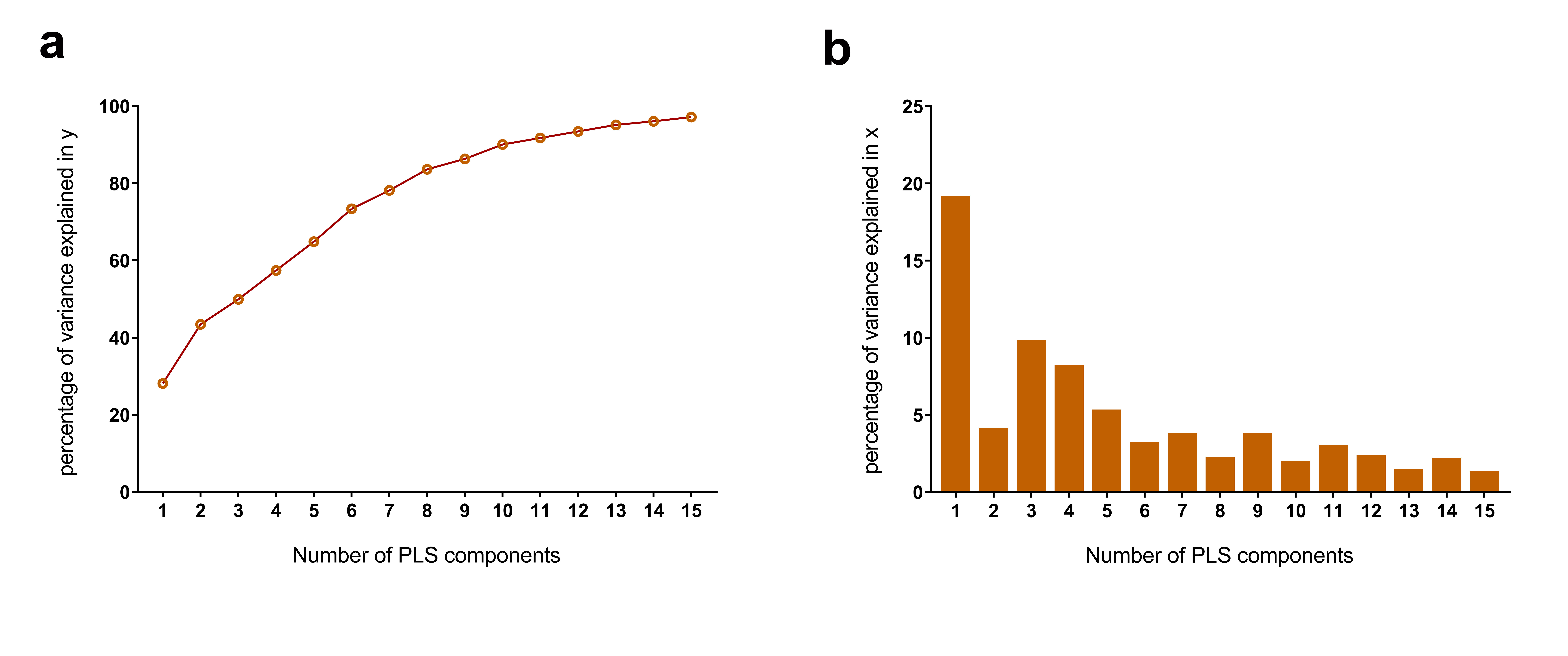

Supplement: Supplementary file 1 — Data S1. [file CNS-30-e14680-s002.zip › Fig. S2.tif]

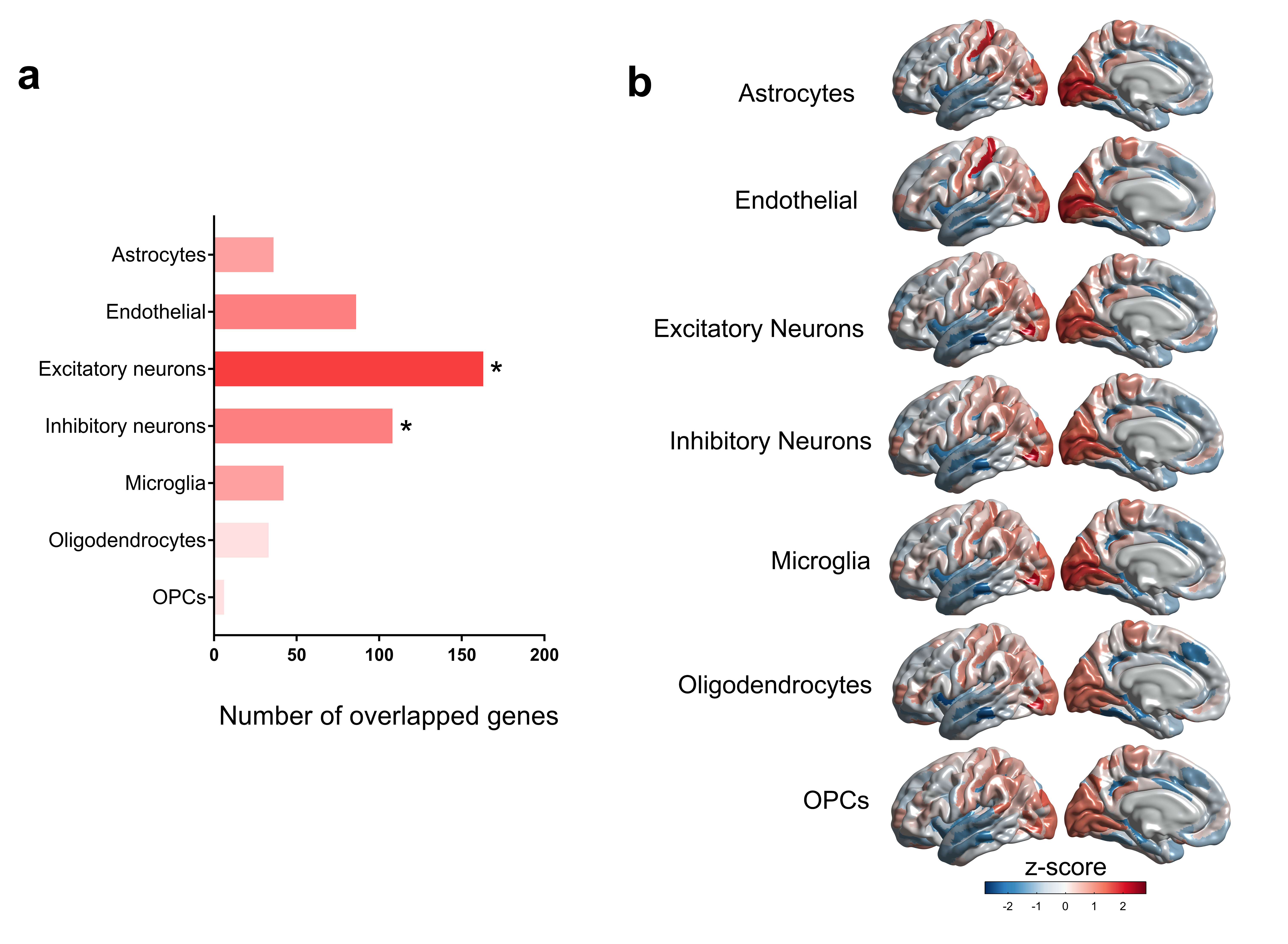

Supplement: Supplementary file 1 — Data S1. [file CNS-30-e14680-s002.zip › Fig. S6.tif]
